# A GFP-tagged version of the pseudorabies virus protein UL56 localizes to the Golgi and *trans*-Golgi network through a predicted C-terminal leucine-rich helix in transfected cells

**DOI:** 10.1186/s12985-019-1191-z

**Published:** 2019-06-20

**Authors:** Chuang Lyu, Xuehui Cai

**Affiliations:** State Key Laboratory of Veterinary Biotechnology, Harbin Veterinary Research Institute of Chinese Academy of Agricultural Sciences, Haping Road No.678, Xiang Fang District, Harbin, 150069 Heilongjiang China

**Keywords:** pUL56, GM130, Co-localization, Rab6a, Transmembrane helix

## Abstract

**Background:**

Pseudorabies virus (PRV) protein UL56 (pUL56) has been implicated in viral dissemination and virulence in vivo. However, the properties of PRV pUL56 remain largely unknown. In the present study, we aim to investigate the subcellular localization of pUL56 and the underlying molecular basis in transfected cells.

**Methods:**

Constructs of N-terminal green fluorescent protein (GFP) fused pUL56 and its truncations were employed for investigating subcellular localization and further identifying amino acids crucial for pUL56 localization in transfected Vero cells. Finally, the identified amino acids were replaced with alanine for confirming if these mutations could impair the specific localization of pUL56.

**Results:**

The pUL56 predominantly localized at the Golgi and *trans*-Golgi network (TGN) through its predicted C-terminal transmembrane helix in transfected Vero cells. A Golgi-associated protein Rab6a, independent of interaction with pUL56, was significantly downregulated by pUL56. Further, we found three truncated pUL56 C-terminal fragments (174–184, 175–185 and 191–195) could restrict GFP in the perinuclear region, respectively. Within these truncations, the ^174^proline (P), ^181^leucine (L), ^185^L and ^191^L were essential for maintaining perinuclear accumulation, thus suggesting an important role of leucine. Alanine (A) mutagenesis assays were employed to generate a series of pUL56 C-terminal mutants on the basis of leucine. Finally, a pUL56 mutant M10 (^174^P/A-^177^L/A-^181^L/A-^185^L/A-^191^L/A-^194^L/A-^195^I/A-^196-197^L/A-^200^L/A) lost Golgi-TGN localization. Thus, our data revealed that the leucine-rich transmembrane helix was responsible for pUL56 Golgi-TGN localization and retention, probably through specific intracellular membrane insertion.

**Conclusion:**

Our data indicated that the C-terminal transmembrane helix was responsible for the Golgi-TGN localization of pUL56. In addition, the leucines within C-terminal transmembrane helix were essential for maintaining pUL56 Golgi-TGN retention in cells. Further, the pUL56 can induce downregulation of Golgi-associated protein Rab6a.

**Electronic supplementary material:**

The online version of this article (10.1186/s12985-019-1191-z) contains supplementary material, which is available to authorized users.

## Background

Pseudorabies virus (PRV), a causative agent of pseudorabies (PR) or Aujezsky’s disease, belongs to the *Herpesviridae* family, *Alphaherpeviridae* subfamily [1]. Other *Alphaherpeviridae* subfamily members include the human pathogens varicella-zoster virus, herpes simplex virus (HSV) 1 and 2, and bovine pathogen bovine herpesvirus 1. Alphaherpesviruses can establish either lytic or latent infections after invading the nervous system [2]. PRV is a double-stranded DNA virus that contains a large genome comprising more than 70 genes [3, 4]. The viral genome is wrapped in an icosahedral capsid surrounded by a proteinaceous tegument layer and a lipid envelope [5]. Tegument proteins in alphaherpesviruses link the viral capsid to the envelope and contribute to multiple biological functions [5]. These proteins can be classified as “inner” or “outer” tegument proteins, on the basis of their association with either the capsid or viral envelope during entry and egress [6, 7].

Recently, PRV protein UL56 (pUL56), a tail-anchored type-II membrane protein, has been identified as a virulent factor that contributes to viral dissemination in the nervous system and to pathogenicity in rodents [8]. Thus, interaction of pUL56 with the host might play an important role in viral pathogenicity. Despite the properties of PRV pUL56 have not been adequately explored, the functions and properties of HSV pUL56 have been reported in previous studies. A deficiency of the HSV1 *UL56* gene attenuates neuro-invasion of the virus in mice but does not affect viral growth ability in vitro [9, 10]. Furthermore, the carboxy (C)-terminal hydrophobic region within pUL56 has been shown to be important for the pathogenicity of HSV1 [11]. In HSV2, pUL56 can target to the Golgi complex and cytoplasmic vesicles in infected and transfected cells, and it probably functions in the vesicular trafficking processes [12, 13]. However, the significant amino acid sequence divergence in pUL56 among HSV1 and 2 and PRV implies potential diversity in the molecular mechanisms underlying the properties and functions of these proteins.

In this work, we identified that the Golgi and *trans*-Golgi network (TGN) were the main sites of PRV pUL56 targeting in transfected Vero cells. Through truncating pUL56 and performing subcellular localization analyses, we found that the C-terminus was essential for targeting and retaining pUL56 in the Golgi-TGN. Within this region, the ^174^proline (P), ^195^ isoleucine (I), and most leucine (L) residues in the predicted transmembrane helix were found to be crucial for maintaining Golgi-TGN localization of pUL56, because replacement of these amino acids by alanine (A) fully abrogated the Golgi-TGN localization of pUL56. Moreover, this molecular basis was found to be conserved in genotype I and II pUL56.

## Methods

### Virus and cell culture

Vero and HEK293T cells were cultured in Dulbecco’s Modified Eagle’s Medium (DMEM; GIBCO, US) supplemented with 10% fetal bovine serum (FBS; GIBCO, US) and 1% penicillin-streptomycin, and kept at 37 °C in an atmosphere of humidified 5% CO_2_. The PRV HeN1 strain was propagated in Vero cells. Viral genome was extracted using an EasyPure Viral DNA/RNA Kit (TransGen Biotech, China) according to the manufacturer’s instructions.

### Antibodies

The following primary antibodies were used: rabbit anti-GM130 polyclonal antibody (11308–1-AP; Proteintech, USA), rabbit anti-EEA1 polyclonal antibody (22266–1-AP; Proteintech, USA), mouse anti-MTC02 monoclonal antibody (ab3298; Abcam, UK), mouse anti-Flag monoclonal antibody (F1804; Sigma-Aldrich), mouse anti-HA monoclonal antibody (H9658; Sigma-Aldrich), mouse anti-GFP monoclonal antibody (66002–1-lg; Proteintech), mouse anti-β-actin monoclonal antibody (A2228; Sigma-Aldrich). The DyLight 800 labeled Goat anti-Mouse IgG (H + L) antibody (KPL, US) was used as secondary antibody for Western blot analyses.

### Amino acid sequence analyses

The amino acid sequences of PRV pUL56 were obtained from GenBank: HeN1 strain (accession No. KP098534), TJ strain (accession No. KJ789182), JS-2012 strain (accession No. KP257591), Becker strain (accession No. JF797219), and Kaplan strain (accession No. KJ717942). Geneious 9.1.4 and DNAstar Protean softwares were used for amino acid sequence alignment and hydrophobic prediction, respectively. The secondary structure of pUL56 was predicted with online tools via the TMHMM Server v.2.0 (http://www.cbs.dtu.dk/services/TMHMM/) and PSIPRED v3.3 (http://bioinf.cs.ucl.ac.uk/psipred/).

### Plasmid construction and transfection

The *UL56* gene was amplified by polymerase chain reaction (PCR) from the extracted viral genome with primers UL56 GFP F/R, UL56 Flag F/R or UL56 HA F/R, respectively (Additional file 2: Table S1). The PCR was performed using KOD FX Neo DNA polymerase (TOYOBO, Japan) according to the manufacturer’s instructions at a final 50 μL reaction volume containing 2× buffer (25 μL), forward primer (2.5 μL; 10 μM), reverse primer (2.5 μL; 10 μM), KOD FX Neo (1.25 μL; 1 unit/μL), dNTP (7.5 μL; 2 mM each), genomic DNA (~ 200 ng) and ddH_2_O. The designated plasmids pAcGFP-UL56, p3 × Flag-UL56 or pCMV-HA-UL56 were constructed by cloning the UL56 PCR product into pAcGFP1-C1, p3 × Flag, or pCMV-HA at indicated restriction sites (Additional file 2: Table S1). The UL56 truncations S1–6 were PCR amplified from pAcGFP-UL56 and cloned into pAcGFP1-C1 by using the primers shown in Additional file 2: Table S1. Complementary oligonucleotides used for constructing shorter UL56 truncations were synthesized and annealed to form a DNA duplex containing cohesive ends, and then cloned into *Xho*I and *Hin*dIII digested pAcGFP1-C1 (Additional file 2: Tables S2-S4).

The Rab6a open reading frame was PCR amplified from HEK293T cDNA using primers shown in Additional file 2: Table S1. The Rab6a PCR product was cloned into pAcGFP1-C1 or p3 × Flag at *Kpn*I/*Bam*HI or *Hin*dIII/*Bam*HI sites. All recombinant plasmids were verified by sequencing.

Transient transfection was performed by using X-tremeGENE HP DNA transfection reagent (Roche, US) according to the manufacturer’s instructions. Briefly, 1 μg plasmid DNA, 2 μL transfection reagent and 100 μL serum-free DMEM were gently mixed and incubated at room temperature (RT) for 20 min, and then evenly dropped onto cells. The transfected cells were incubated for 24 or 48 h for further assays.

### Construction of pUL56 C-terminal mutants by alanine mutagenesis

Overlapping extension PCR-based site-directed alanine mutagenesis was used to generate a series of pUL56 C-terminal mutants. Thirteen pUL56 mutants were constructed by using overlapping primers or oligos containing the required mutations (Additional file 2: Tables S5 and S6). The pUL56 mutants M1~5 and 11~13 were constructed by using overlapping primers containing mutant sites (Additional file 2: Table S5). In UL56 M1 as an example, the forward primer UL56 GFP F and reverse primer M1 R were used for amplifying the first fragment, and then the forward primer M1 F and reverse primer UL56 GFP R were used for amplifying the second fragment with pAcGFP-UL56 as a template. Fragments 1 and 2 were then used as mixed templates for amplifying UL56 M1 by using primers UL56 GFP F and R. The amplified UL56 M1 PCR product was digested with *Xho*I and *Kpn*I and cloned into the pAcGFP1-C1. To obtain UL56 M2~5 and 11~13, the same method was used, and the latter mutant was constructed by using the previous one as a template. UL56 M6 and M9 were constructed by using the forward primer UL56 GFP F and reverse primer containing the mutant site, and the termination codon TGA was followed by a *Kpn*I site. UL56 M7, 8 and 10 were constructed by using the same method as described for the M1 construct except that the second fragment was mutant sites induced oligo (Additional file 2: Table S6).

### Indirect immunofluorescence assay and confocal microscopy

Vero cells seeded in 20 mm glass-bottom cell culture dishes (NEST, China) were transiently transfected with the indicated plasmids. At 24 or 48 h post transfection (hpt), the transfected cells were rinsed with phosphate-buffered saline (PBS) three times and then fixed in 4% paraformaldehyde for 1 h at 4 °C. The cells were rinsed with PBS, and dishes were blocked in 3% bovine serum albumin (BSA) at 37 °C for 1 h. The cells were incubated with anti-GM130, −MTC02, −EEA1 or -Flag antibodies at 4 °C overnight; rinsed three times with PBS; and incubated for 1 h with Alexa Fluor 568-labeled goat anti-rabbit or mouse IgG antibodies (Thermo Fisher, USA) diluted in PBS at RT. Then, the cellular nuclei were stained with 4′,6-Diamidino-2-phenylindole (DAPI, Invitrogen) for 10 min at RT. The cells were rinsed and then imaged with an LSM800 confocal microscope equipped with a 63× oil immersion objective (Zeiss). All primary antibodies were 1:500 diluted with buffer containing 0.3% Triton X-100 (Sigma-Aldrich), 1% BSA and 0.01% sodium azide (Sigma-Aldrich) in PBS.

### Coimmunoprecipitation (co-IP)

HEK293T cells grown to 80% confluence in 6-well plates were co-transfected with p3 × Flag-Rab6a and pAcGFP or pAcGFP-UL56 (1.5 μg for each plasmid). At 24 hpt, the transfected cells were harvested and lysed in NP40-lysis buffer (Beyotime Biotechnology, China) containing 1 mM PMSF and complete protease inhibitor cocktail (Sigma-Aldrich) for 1 h on ice. Cell lysates were centrifuged (12,000 rpm, 10 min, 4 °C) and obtained supernatants were used for immunoprecipitation with FLAG M2 beads (Sigma-Aldrich), according to the manufacturer’s instructions. Briefly, the supernatants were incubated with lysis buffer pre-washed FLAG M2 beads and incubated for 6 h under continuous shaking at 4 °C. The beads were rinsed with PBS (pH 7.4) for three times, and immunoprecipitated proteins were separated by sodium dodecyl sulfate-polyacrylamide gel electrophoresis and subjected to Western blot analyses.

### Statistical analysis

The results were expressed as mean ± SEM. Statistical analysis was performed using GraphPad Prism 6.01 software (GraphPad Software, Inc.). Quantification of bands in Western blot was performed by densitometry using Image J software. The density for Flag-Rab6 was normalized to that of β-actin. The difference was analyzed by unpaired two-tail Student’s *t*-test based on three independent replicates. *P* < 0.05 was taken as the criterion for statistical significance.

## Results

### Genotype I and II PRV pUL56 contains identical C-terminal transmembrane helices

**Fig. 1:**
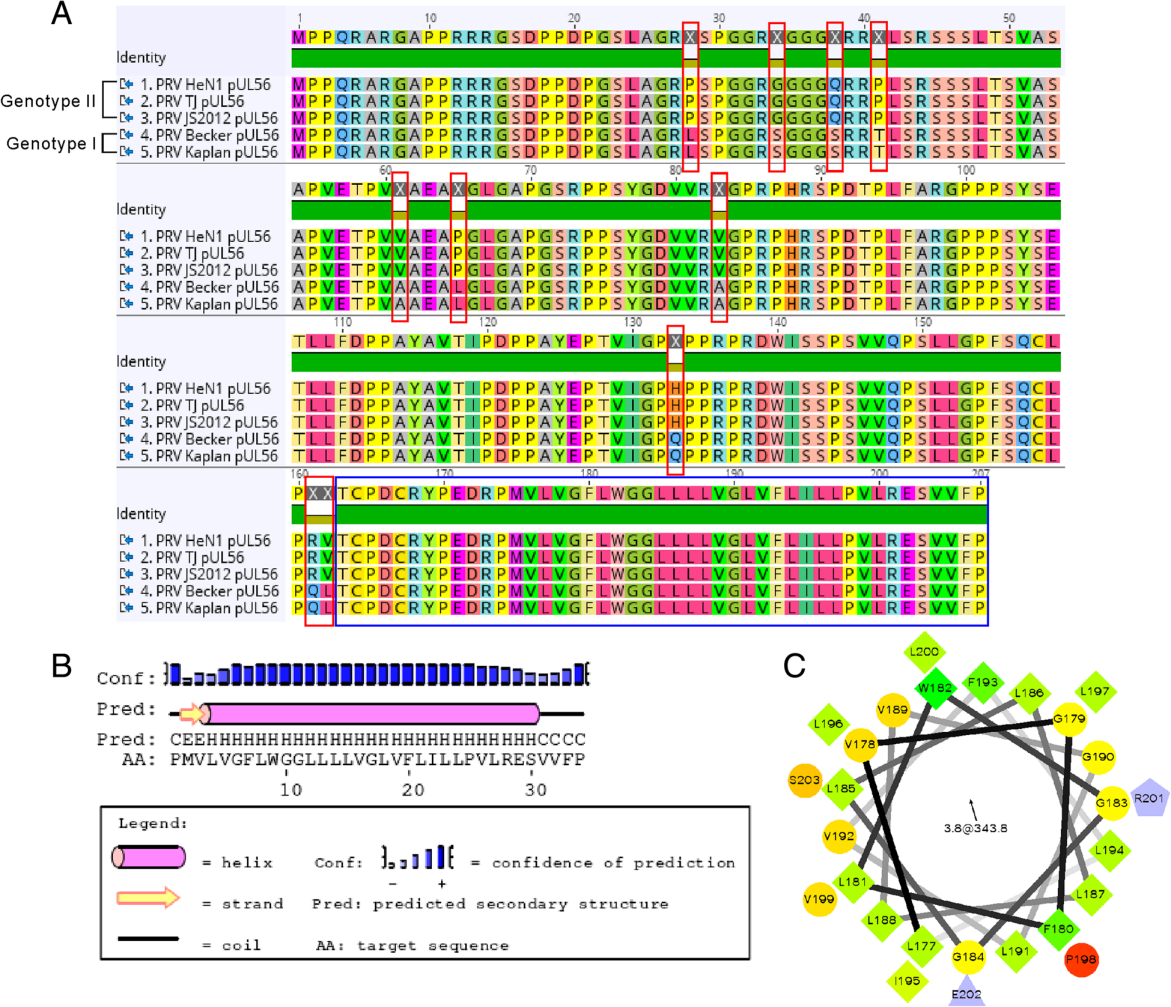
Genotype I and II PRV pUL56 contains identical C-terminal transmembrane helices. **a** Alignment of pUL56 sequences between genotype I and II PRV strains shows 44 identical amino acids (163–207) at the C terminus (blue rectangle). Differential amino acids are indicated by red rectangles. **b** The 177–203 aa in pUL56 is identified as a helix structure, as predicted by PSIPRED. **c** Helical wheel displaying a hydrophobic character of the helix

An emergence of PRV variants in China results in classification of PRV into two genotypes (I and II) [14, 15]. The failure of Bartha-K61 vaccination results in outbreak of genotype II PRV in China since 2011 [4, 15]. In order to compare difference between genotype I and II PRV pUL56, the amino acid sequences collected from five strains were used for further analyses. A sequence alignment showed a conserved C-terminus in both genotypes, although amino acid differences were also present in other regions of pUL56 (Fig. 1a). Furthermore, the TMHMM and PSIPRED protein sequence analyses indicated a predicted transmembrane helix (177–203) at the C-terminus (Fig. 1b and Additional file 1: Figure S1A). Plotted helical wheel diagram for the predicted helix revealed a hydrophobic characteristic (Fig. 1c).

### pUL56 localizes to the Golgi-TGN in transfected Vero cells

**Fig. 2:**
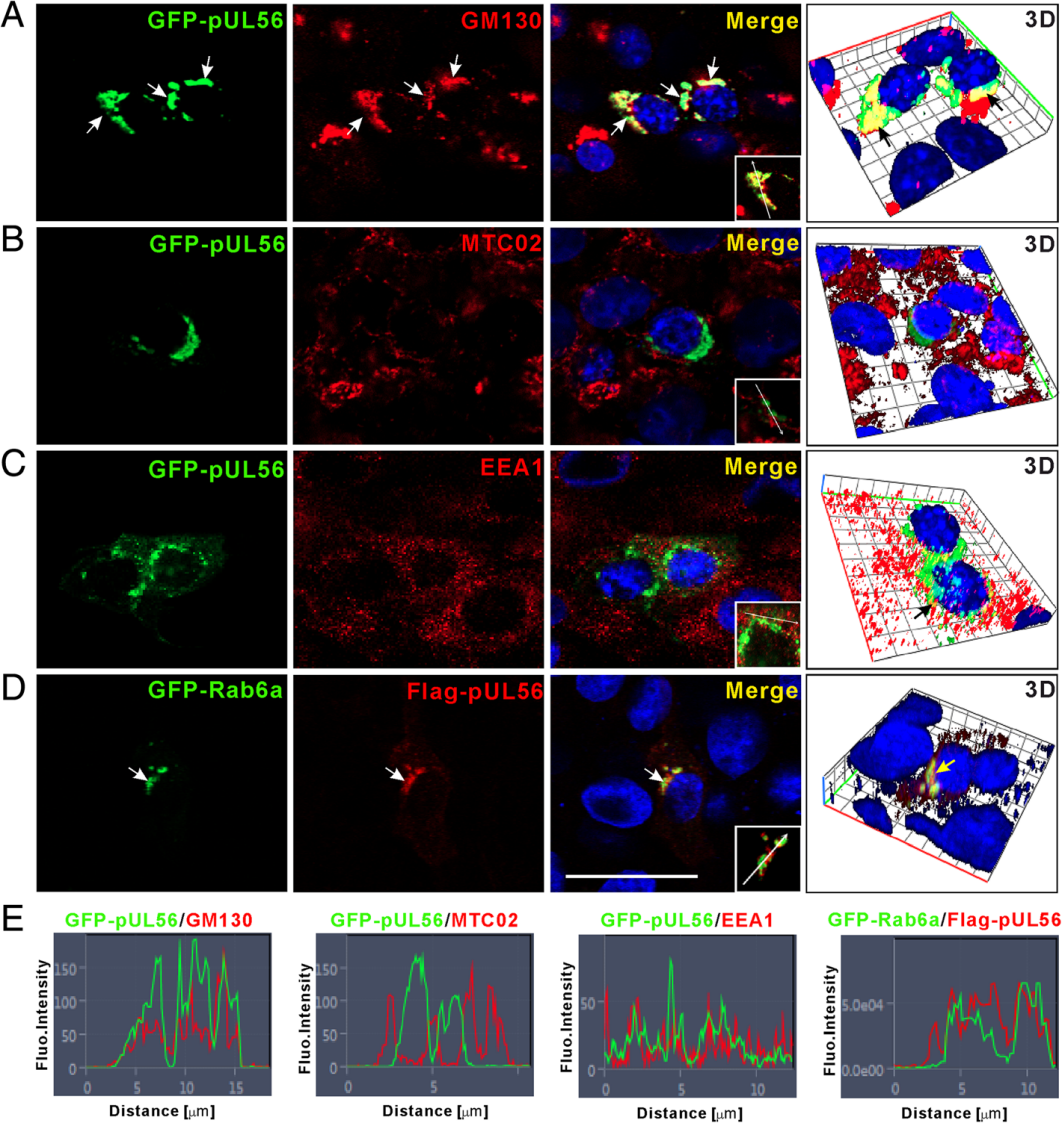
Subcellular localization of pUL56 in transfected Vero cells. **a** The overexpressed GFP-pUL56 distributes in the perinuclear region and co-localizes with the *cis*-Golgi marker GM130 (arrows). **b** The GFP-pUL56 does not co-localize with the mitochondrial marker MTC02. **c** The GFP-pUL56 shows partial co-localization with the early endosome marker EEA1 in the cytoplasm. **d** The Flag-pUL56 co-localizes with GFP-Rab6a in the co-transfected cells (arrows). **e** Co-localization analyses of pUL56 with each marker alone the plotting line shown in (**a**-**d**). Nuclei are stained with DAPI. Scale bar indicates 25 μm (**a**-**d**)

To investigate the subcellular localization of in vitro overexpressed pUL56, the plasmid pAcGFP-UL56 was transfected into Vero cells. At 24 hpt, the expressed GFP-pUL56 was observed mainly at the perinuclear region in a typical organelle localization pattern in addition to a punctate distribution in the cytoplasm (Fig. 2). Subsequently, the GFP-pUL56-expressing cells were stained with anti-GM130, anti-MTC02 or anti-EEA1 antibodies for specially labelling endogenous *cis*-Golgi, mitochondria, and early endosome, respectively. Confocal micrographs showed that the GFP-pUL56 partly co-localized with GM130, but not with MTC02, and some GFP-pUL56 puncta co-localized with EEA1 in the cytoplasm (Fig. 2a-c and e). To further identify the Golgi compartment localization of pUL56, we constructed a plasmid expressing GFP-Rab6a, a small GTPase, which is a ubiquitous rab associated with membranes of the Golgi complex as well as that of trans-Golgi network (TGN) [16]. Co-expression of GFP-Rab6a with Flag-pUL56 showed a strong co-localization in the co-transfected Vero cells (Fig. 2d and e). Taken together, these results demonstrate that pUL56 localizes to Golgi-TGN.

### The Golgi-associated protein Rab6a is downregulated by pUL56

**Fig. 3:**
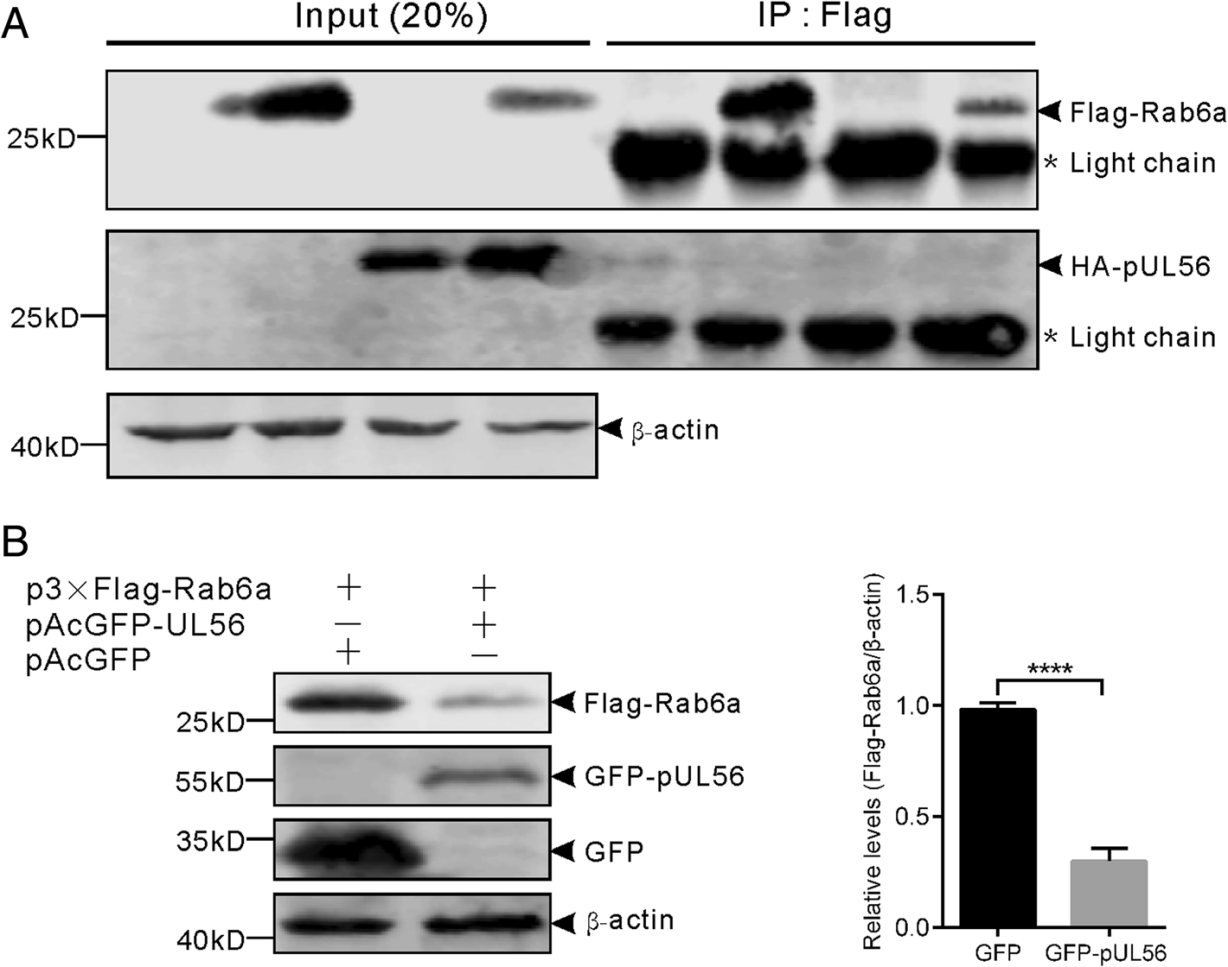
The pUL56 induces Golgi-associated protein Rab6a downregulation. **a** Co-IP assay shows that pUL56 does not interact with Rab6a. **b** HEK293T cells were co-transfected with plasmid expressing Flag-Rab6a with GFP or GFP-pUL56 (1 μg per plasmid). At 48 hpt, the cell lysates were collected and subjected to Western blot analysis using antibodies against Flag, GFP and β-actin. The optical density ratio of Flag-Rab6a/β-actin in GFP expressing group was set to 1. ****, *P* < 0.0001

Next, we sought to examine whether pUL56 interacted with Rab6a, due to pUL56 well co-localized with Rab6a at Golgi-TGN. Co-IP assay was employed to examine interaction between pUL56 and Rab6a. The result showed that pUL56 could not interact with Rab6a (Fig. 3a). Thus, this data has excluded the possibility that pUL56 localizes to Golgi-TGN through interaction with Rab6a.

A constant amount of plasmid p3 × Flag-Rab6a was co-transfected with pAcGFP or pAcGFP-UL56 into HEK293T cells, the protein level of Flag-Rab6a was detected by Western blot with anti-Flag antibody at 48 hpt. Data from three independent experiments showed that Flag-Rab6a was significantly downregulated by GFP-pUL56 as compared to GFP, thus indicating Rab6a, independent of interaction with pUL56, can be significantly downregulated by pUL56. (Fig. 3b).

### The predicted C-terminal transmembrane domain is crucial for pUL56 intracellular membrane insertion

**Fig. 4:**
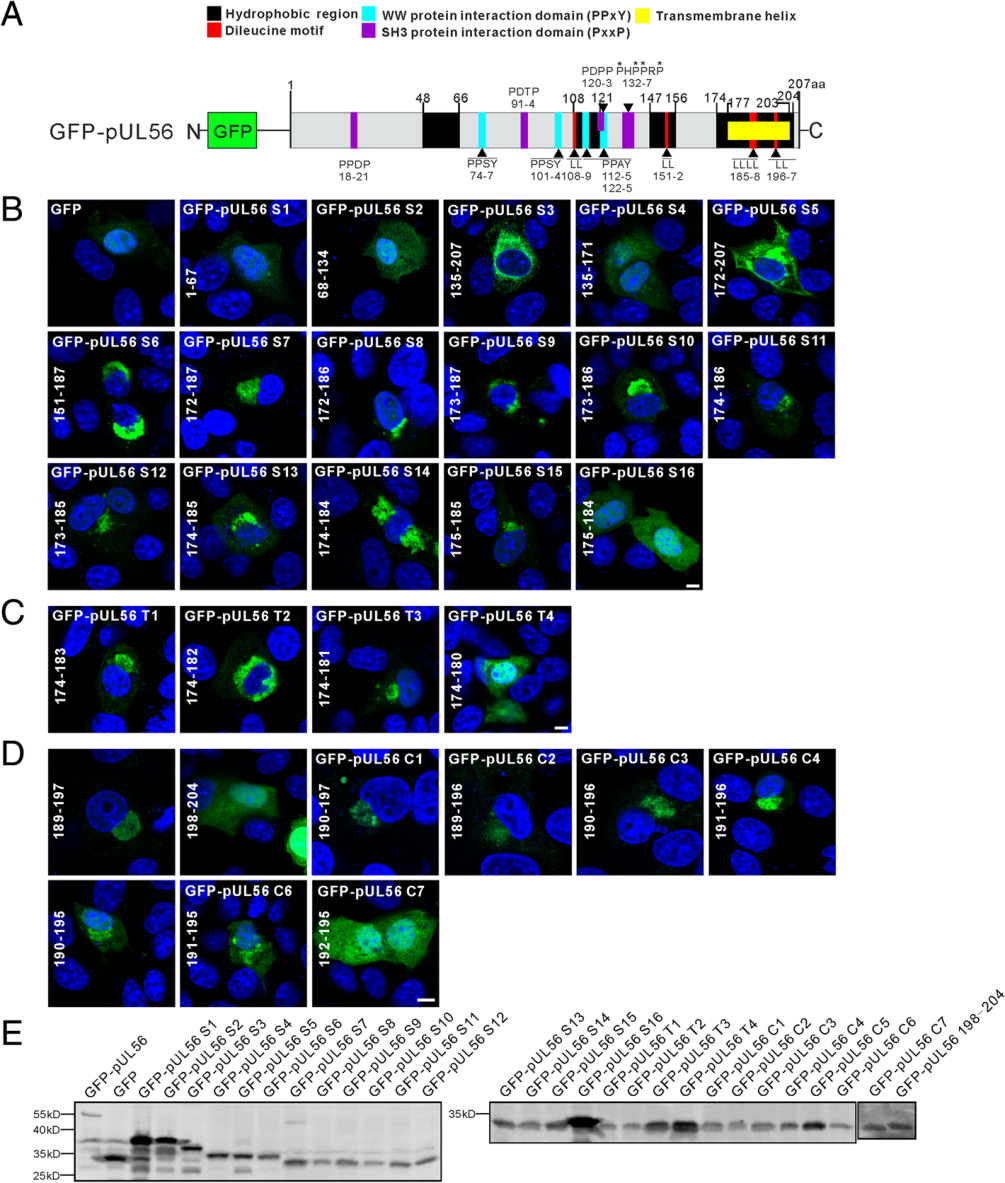
Subcellular distribution of pUL56 truncations in transfected Vero cells. **a** Schematic representation of pUL56 fused with GFP at the N-terminus. Five functional domains/motifs including hydrophobic region (48–66, 108–121, 147–156, and 174–204 aa), PPxY motifs (74–77, 101–104, 112–115, and 122–125 aa), PxxP motifs (18–21, 91–94, 120–123, and 132–137 aa) and one predicted transmembrane helix (177–203 aa) are labelled with different colors. **b**-**d** Representative confocal micrographs show subcellular distribution of pUL56 truncations in transfected Vero cells. **e** Western blot shows the molecular weight of GFP fused WT pUL56 and its truncations. Scale bar indicates 5 μm (**b**-**d**)

Amino acid sequence analyses showed at least three sets of motif embedded in pUL56, including WW-domain protein interaction domain (PPxY motif; here and below, “x” indicates any residue), di-leucine (LL) motif, and SH3-domain protein interaction domain (PPxP motif) (Fig. 4a) [17–19]. In addition, four predicted hydrophobic regions (48–66, 108–121, 147–156, and 174–204) diffusely distributed within pUL56 (Fig. 4a and Additional file 1: Figure S1B). The full-length pUL56 was first truncated into three fragments, including S1 (1–67), S2 (68–134) and S3 (135–207). The subcellular distribution showed S1 and S2 were throughout cells, with a similar distribution to that of GFP, whereas S3, a transmembrane helix containing segment, largely accumulated in the cytoplasm (Fig. 4b). In addition, the GFP fused S3 has a smaller molecular weight than S1 and S2 as shown in Fig. 4e, thus indicating an intracellular membrane insertion.

To analyze the crucial amino acid underlying intracellular membrane insertion of S3, the S3 was truncated into the shorter fragments S4 (135–171), S5 (172–207) and S6 (151–187). The S4 was observed throughout cells; however, S5 and S6 accumulated at perinuclear region, thus indicating that the overlapped region designated S7 (172–187) between S5 and S6 might contain intracellular membrane insertion information (Fig. 4b). Then, the S7 was truncated by single amino acid deletion from the C- or/and N-terminus, resulting in truncations S8 (172–186), S9 (173–187), S10 (173–186), S11 (174–186), S12 (173–185), S13 (174–185), S14 (174–184), S15 (175–185), and S16 (175–184). Subcellular distribution of these truncations showed that S8-S15 accumulated at perinuclear region, but S16 was observed throughout cells (Fig. 4b). Therefore, the presence of either N-terminal ^174^P or C-terminal ^185^L in S16 could rescue the intracellular membrane insertion ability.

To clarify the crucial amino acids required for maintaining the perinuclear distribution of pUL56 within fragment 174–185, we further truncated S13 into shorter fragments T1 (174–183), T2 (174–182), T3 (174–181), T4 (174–180) by single amino acid deletion from the C-terminus. Perinuclear accumulation was observed in T1–3-expressing cells, whereas T4 was exclusively distributed throughout the cells (Fig. 4c). Together, these data indicated that ^174^P, ^181^L, and ^185^L are crucial for maintaining intracellular insertion within either fragment 174–184 or 175–185.

Using a similar strategy, we continued to investigate whether the remaining amino acids (189–204) within the C-terminal transmembrane helix were also responsible for the intracellular membrane insertion. Analysis of subcellular distribution showed that 189–197 and its truncations C1–6, but not 198–204 or C7, accumulated at perinuclear region. Thus, the presence of N-terminal ^191^L in C6 contributes to perinuclear accumulation (Fig. 4d).

Furthermore, we analyzed the protein molecular weight for each truncation using Western blot (Fig. 4e). The result showed that S7–16, T1–4, and C1–7 had approximated protein molecular weight, thus indicating protein passive transport could not influence specific perinuclear distribution of each truncation, probably because a specific intracellular membrane insertion.

Of note, all confocal micrographs shown in Fig. 4 represent the predominant subcellular distribution pattern (> 70% ratio in all transfected cells, around 100 cells were counted) for each pUL56 truncation.

### A leucine-rich helical structure within the C-terminus provides a molecular basis for pUL56 Golgi-TGN localization

**Fig. 5:**
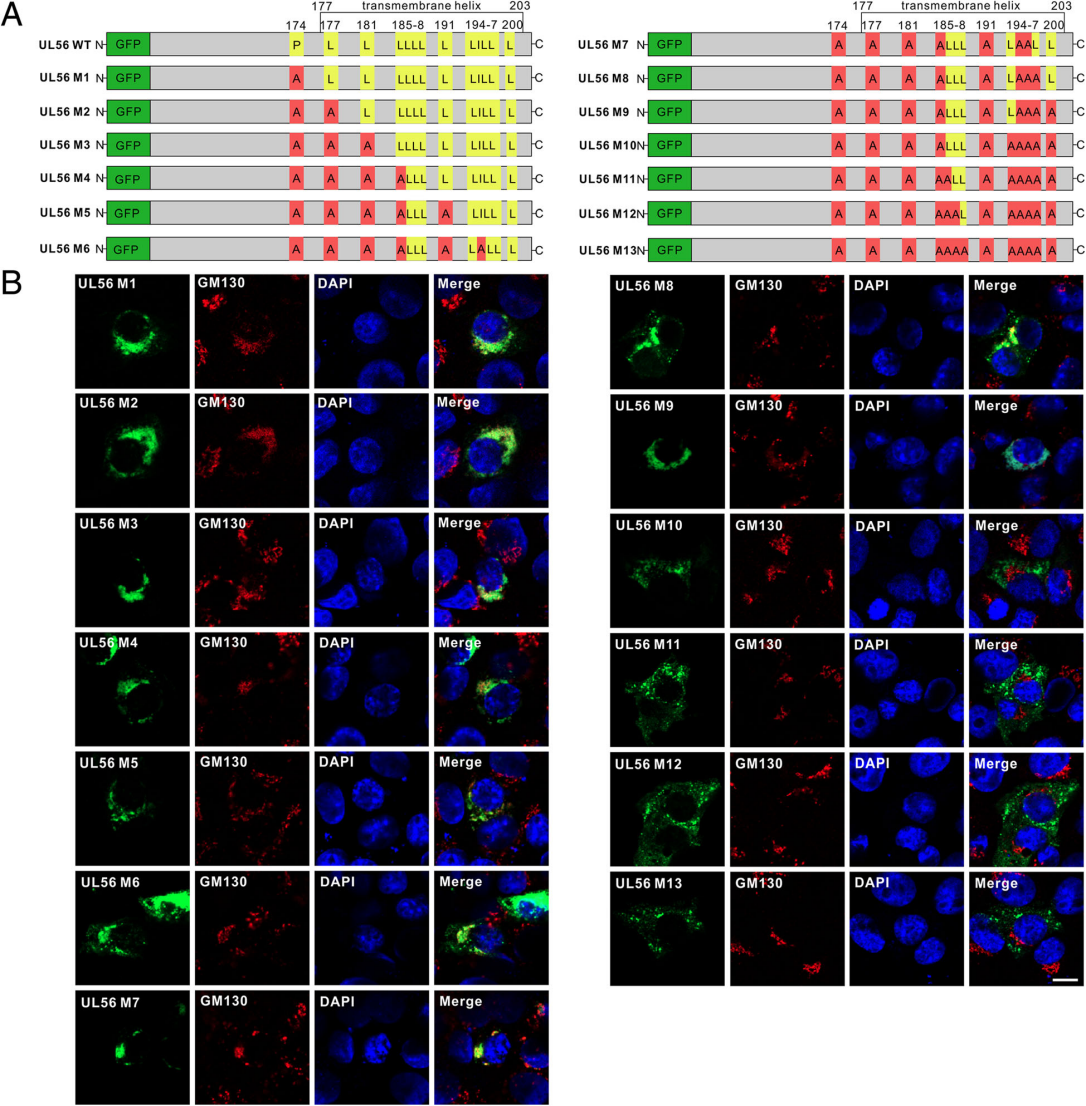
Alanine mutagenesis within the C-terminal transmembrane helix in pUL56 disrupts Golgi localization. **a** Schematic shows site-directed alanine mutagenesis in pUL56. **b** Confocal micrographs show the effects of pUL56 C-terminal alanine mutagenesis on Golgi localization in transfected Vero cells. Scale bar indicates 10 μm (B)

We hypothesize the leucine within pUL56 C-terminus plays an important role in its Golgi-TGN localization, because the leucine seems to be the crucial residue affecting the pUL56 truncation intracellular membrane insertion. To identify this hypothesis, we constructed contiguous pUL56 mutants based on the leucine as shown in Fig. 5a. Subsequently, the Golgi localization of these mutants was evaluated by confocal microscopy using GM130 as a Golgi marker. The co-localization analyses showed that the pUL56 mutants M1–8 largely localized at Golgi complex, however, the M9–13 localized in cytoplasm, thus indicating loss of intracellular membrane-tethered ability.

Thus, we concluded that the leucine-rich helical structure within the C-terminus contributes to pUL56 Golgi-TGN localization and retention, probably through maintaining intracellular membrane insertion ability.

## Discussion

In view of the importance of PRV pUL56 in viral virulence and dissemination in the rodent nervous system [8], it is essential to understand the properties of pUL56. In the current study, we performed a series of experiments to characterize pUL56 subcellular localization and to clarify the underlying molecular basis. To achieve this goal, we fuse a GFP tag at the N terminus of pUL56 due to a predicted transmembrane is present at the C terminus. Utilizing GFP as an indicator for investigating subcellular localization related signals in virus or host proteins of interest has been successfully applied in previous reports [20–24].

Although pUL56 has a transmembrane character, the in vitro expressed GFP-pUL56 is not observed on the plasma membrane. In contrast, pUL56 is more likely to be an intracellular membrane-tethered type-II membrane protein, owing to its particular organelle localization. Co-localization analyses indicated that the pUL56 localized to Golgi complex and TGN, in addition to showing a cytoplasmic punctate distribution. The punctate pUL56 showed partial co-localization with the early endosome marker EEA1, thus suggesting that some of the cytoplasmic pUL56 could be transported by the endocytic membrane transport pathway induced through fusion with early endosomes, a process probably involved in the vesicular transport [25, 26].

Rab6a, the most abundant Golgi-associated Rab protein, is involved in maintenance of Golgi organization and different Golgi-associated trafficking pathways, such as Golgi-derived vesicle transport, in eukaryotic cells [27–32]. Our finding that pUL56 induced remarkable downregulation of Rab6 might, at least partly, explain another observation showing abnormal Golgi organization in Golgi-localized WT and mutant pUL56 expressing cells as compare with control ones (Figs. 2 and 5).

There are three sets of well characterized motifs in pUL56 (Fig. 4a). Among them, the dileucine motif is of high interest because its importance in targeting transmembrane proteins to the endosome/lysosome system and TGN [33–38]. Although several di-leucine motifs are present in PRV pUL56, our data showed that none had the ability to target pUL56 to the TGN. In addition, a recent finding indicates that PRV pUL56 interacts with swine leukocyte antigen class I molecules and targets them for degradation through lysosome pathway in cells [39]. Thus, the dileucine motif in pUL56 might act a role in targeting to the late endosome/lysosome. These findings imply that targeting of pUL56 interacting host proteins to lysosomes for degradation can be a common event that occurs during PRV infection.

Although the amino acid sequences of pUL56 in α-herpesviruses HSV1 and 2 and PRV display quite low homology (data not shown), the HSV2 pUL56 has also been shown to localize to the Golgi complex and cytoplasmic vesicles [13]. This cytoplasmic vesicle localization was identified by co-localization analyses with EEA1. Additionally, one study has indicated that HSV2 pUL56 interacts with the kinesin motor protein KIF1A which is a Rab6 interactor [16, 40, 41]. A family of coiled-coil-rich proteins named golgins, including golgin-230/245/256 and golgin-97, have been reported to target to the Golgi complex through a C-terminal domain containing a conserved tyrosine residue binding to Rab6 [42]. On the basis of these findings, HSV2 pUL56 could localize to the Golgi complex, at least partly, through a contiguous pUL56-KIF1A-Rab6 interaction based on the tyrosine residue within N-terminal amino acid 20–36 of pUL56. However, this scenario cannot be used for explaining the specific Golgi localization of PRV pUL56 based on two findings in this paper: 1) the UL56 N-terminal truncation S1 distributes throughout cells; 2) PRV pUL56 can mediate remarkable degradation of Rab6a. Thus, these data suggest difference between PRV and HSV on the mechanism of pUL56 Golgi localization.

Consequently, we identified a minimum site-directed mutation which caused a shift from Golgi localization into cytoplasm in pUL56 M10. In general, the localization and retention of protein in Golgi components can be achieved via multiple mechanisms, including protein-protein interactions, affinity between proteins and the Golgi membrane-lipid environment, and affinity between proteins and the transport vesicle coat protein complex I/II [22, 43–46]. Accordingly, the Golgi localization of pUL56 is likely to be due to the specific interaction between hydrophobic residues and Golgi phospholipid groups, on the basis of its 3D conformation induced by the leucine-rich transmembrane helix.

In conclusion, we have elucidated the molecular basis underlying PRV pUL56 Golgi-TGN localization. In addition, a Golgi-associated protein Rab6a is found to be downregulated by pUL56, which may result in Golgi ribbon fragmentation, and further affecting PRV assembly. These results shed new light on the alphaherpesvirus pUL56 Golgi localization, in particular through the transmembrane helix, in eukaryotic cells.

## Conclusion

In the current study, the PRV pUL56 was found to anchor to Golgi-TGN in the transfected cells. The sole transmembrane helix at the C-terminus of pUL56 contributed to the Golgi-TGN localization. Further investigations showed that the leucine within the transmembrane was crucial for sustaining pUL56 Golgi retention. Another finding showed Golgi-associated Rab6a was significantly downregulated by pUL56, thus suggesting a possible influence of pUL56 on Golgi structure.

## Additional files

Additional file 1: **Figure S1.** Transmembrane domain prediction and hydrophobic analysis of pUL56. (A) One transmembrane helix in pUL56 is predicated using TMHMM. (B) Hydrophobic domain in pUL56 is analyzed with DNAstar Protean. (TIF 5019 kb)

Additional file 2: **Table S1.** Primers used in this study. **Table S2.** Oligos used in this study 1. **Table S3.** Oligos used in this study 2. **Table S4.** Oligos used in this study 3. **Table S5.** Primers used in alanine mutagenesis assays. **Table S6.** Oligos used in alanine mutagenesis assays. (DOCX 31 kb)

## Data Availability

Not applicable.

## Abbreviations

BSA: Bovine serum albumin
Co-IP: Coimmunoprecipitation
DMEM: Modified Eagle’s Medium
FBS: Fetal bovine serum
GFP: Green fluorescent protein
HSV: Herpes simplex virus
PBS: Phosphate-buffered saline
PCR: Polymerase chain reaction
PRV: Pseudorabies virus
RT: Room temperature
TGN: *trans*-Golgi network
