# Supplementary figures and images for: A GFP-tagged version of the pseudorabies virus protein UL56 localizes to the Golgi and trans-Golgi network through a predicted C-terminal leucine-rich helix in transfected cells
Source: Virol J. 2019 Jun 20;16:81. doi: 10.1186/s12985-019-1191-z (PMC6585060; doi:10.1186/s12985-019-1191-z)

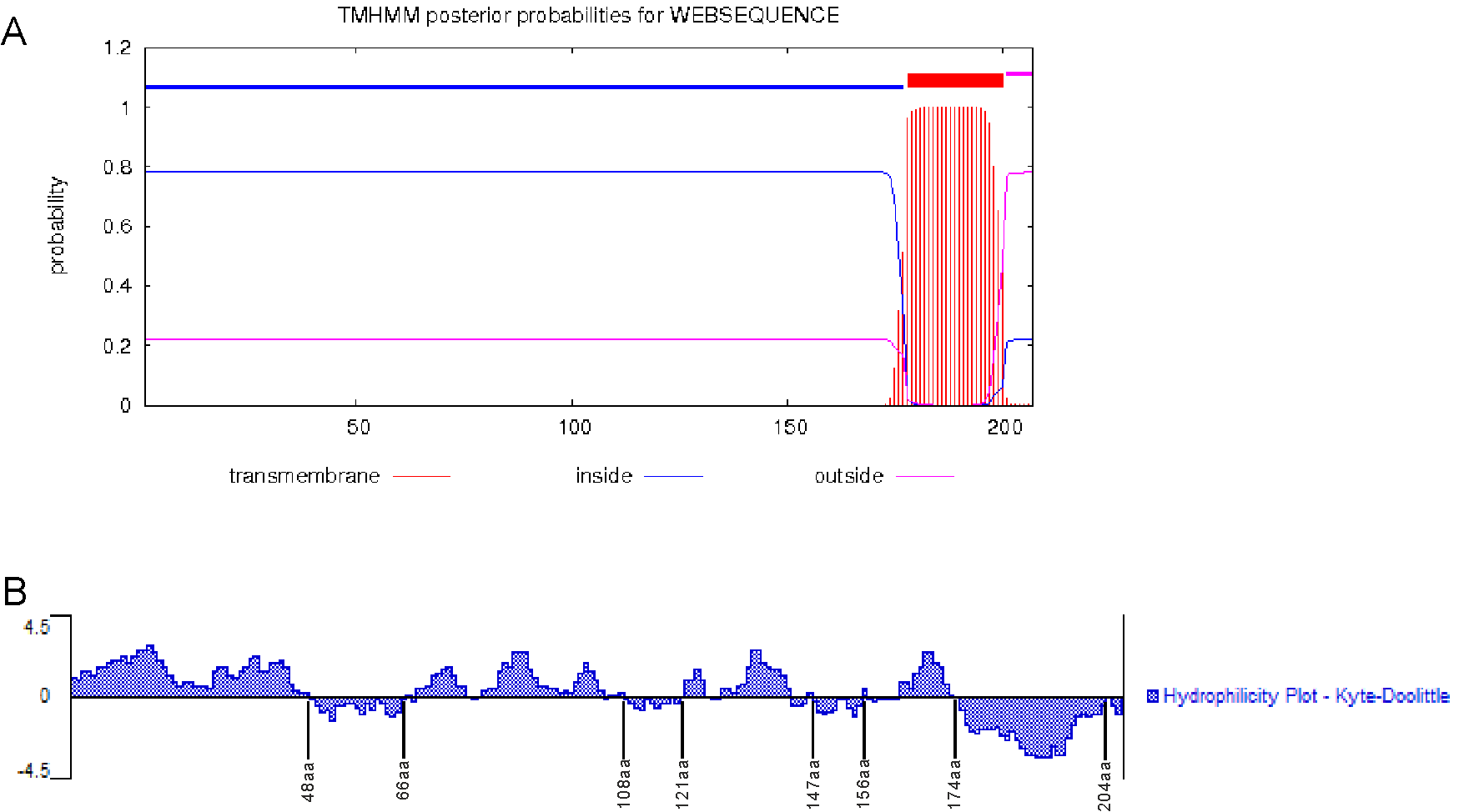

Supplement: Supplementary file 1 — Figure S1. Transmembrane domain prediction and hydrophobic analysis of pUL56. (A) One transmembrane helix in pUL56 is predicated using TMHMM. (B) Hydrophobic domain in pUL56 is analyzed with DNAstar Protean. (TIF 5019 kb) [file 12985_2019_1191_MOESM1_ESM.tif]
