# Supplementary material for: A GFP-tagged version of the pseudorabies virus protein UL56 localizes to the Golgi and trans-Golgi network through a predicted C-terminal leucine-rich helix in transfected cells
Source: Virol J. 2019 Jun 20;16:81. doi: 10.1186/s12985-019-1191-z (PMC6585060; doi:10.1186/s12985-019-1191-z)
Supplement: Supplementary file 2 — Table S1. Primers used in this study. Table S2. Oligos used in this study 1. Table S3. Oligos used in this study 2. Table S4. Oligos used in this study 3. Table S5. Primers used in alanine mutagenesis assays. Table S6. Oligos used in alanine mutagenesis assays. (DOCX 31 kb) [file 12985_2019_1191_MOESM2_ESM.docx]

**Table S1. Primers used in this study**

| Primers | Region (aa) | Sequences (5’-3’) | Vectors |
| --- | --- | --- | --- |
| UL56 GFP F | 1-207 | GC*CTCGAG*CTATGCCTCCACAACGAGCCCGC (*Xho* I) | pAcGFP1-C1 |
| UL56 GFP R  UL56 Flag F  UL56 Flag R  UL56 HA F  UL56 HA R  Rab6a GFP F  Rab6a GFP R  Rab6a Flag F  Rab6a Flag R | 1-207  1-207  1-208  1-208 | GC*GGTACC*TCAGGGAAACACGACGGACTC (*Kpn* I)  GC*AAGCTT*ATGCCTCCACAACGAGCCCGC (*Hin* dIII)  GC*GAATTC*TCAGGGAAACACGACGGACTC (*Eco* RI)  GC*GAATTC*TCATGCCTCCACAACGAGCCCGCG (*Eco* RI)  GC*GGTACC*TCAGGGAAACACGACGGACTC (*Kpn* I)  GC*GGTACC*ATGTCCACGGGCGGAGACTTC (*Kpn* I)  GC*GGATCC*TTAGCAGGAACAGCCTCCTTC (*Bam* HI)  GC*AAGCTT*ATGTCCACGGGCGGAGACTTC (*Hin* dIII)  GC*GGATCC*TTAGCAGGAACAGCCTCCTTC (*Bam* HI) | p3×Flag  pCMV-HA  pAcGFP1-C1  p3×Flag |
| UL56 S1 F | 1-67 | GC*CTCGAG*CTATGCCTCCACAACGAGCCCGC (*Xho* I) | pAcGFP1-C1 |
| UL56 S1 R |  | GC*GGTACC*TCAGAGCCCCGGAGCCTCCGC (*Kpn* I) |  |
| UL56 S2 F | 68-134 | GC*CTCGAG*CTGGCGCCCCCGGCTCCAGGCCG (*Xho* I) | pAcGFP1-C1 |
| UL56 S2 R |  | GC*GGTACC*TCACGGGTGCGGCCCGATGAC (*Kpn* I) |  |
| UL56 S3 F | 135-207 | GC*CTCGAG*CTCCGCGCCCCCGCGACTGGATC (*Xho* I) | pAcGFP1-C1 |
| UL56 S3 R |  | GC*GGTACC*TCAGGGAAACACGACGGACTC (*Kpn* I) |  |
| UL56 S4 F | 135-171 | GC*CTCGAG*CTCCGCGCCCCCGCGACTGGATC (*Xho* I) | pAcGFP1-C1 |
| UL56 S4 R |  | GC*GGTACC*TCATTCGGGGTAGCGGCAGTC (*Kpn* I) |  |
| UL56 S5 F | 172-207 | GC*CTCGAG*CTGACCGCCCGATGGTGCTCGTG(*Xho* I) | pAcGFP1-C1 |
| UL56 S5 R |  | GC*GGTACC*TCAGGGAAACACGACGGACTC (*Kpn* I) |  |
| UL56 S6 F | 151-187 | GC*CTCGAG*CTCTGCTGGGCCCCTTCAGCCAG (*Xho* I) | pAcGFP1-C1 |
| UL56 S6 R |  | GC*GGTACC*TCACAGGAGCAGTCCCCCCCA (*Kpn* I) |  |

**Table S2. Oligos used in this study 1**

| Oligos | Region (aa) | Sequences (5’-3’) | Vectors |
| --- | --- | --- | --- |
| S7 F | 172-187 | *TCGAG*CTGACCGCCCGATGGTGCTCGTGGGCTTCCTCTGGGGGGGACTGCTCCTGTGA*A* | pAcGFP1-C1 |
| S7 R |  | *AGCTT*TCACAGGAGCAGTCCCCCCCAGAGGAAGCCCACGAGCACCATCGGGCGGTCAG*C* |  |
| S8 F | 172-186 | *TCGAG*CTGACCGCCCGATGGTGCTCGTGGGCTTCCTCTGGGGGGGACTGCTCTGA*A* | pAcGFP1-C1 |
| S8 R |  | *AGCTT*TCAGAGCAGTCCCCCCCAGAGGAAGCCCACGAGCACCATCGGGCGGTCAG*C* |  |
| S9 F | 173-187 | *TCGAG*CTCGCCCGATGGTGCTCGTGGGCTTCCTCTGGGGGGGACTGCTCCTGTGA*A* | pAcGFP1-C1 |
| S9 R |  | *AGCTT*TCACAGGAGCAGTCCCCCCCAGAGGAAGCCCACGAGCACCATCGGGCGAG*C* |  |
| S10 F | 173-186 | *TCGAG*CTCGCCCGATGGTGCTCGTGGGCTTCCTCTGGGGGGGACTGCTCTGA*A* | pAcGFP1-C1 |
| S10 R |  | *AGCTT*TCAGAGCAGTCCCCCCCAGAGGAAGCCCACGAGCACCATCGGGCGAG*C* |  |
| S11 F | 174-186 | *TCGAG*CTCCGATGGTGCTCGTGGGCTTCCTCTGGGGGGGACTGCTCTGA*A* | pAcGFP1-C1 |
| S11 R |  | *AGCTT*TCAGAGCAGTCCCCCCCAGAGGAAGCCCACGAGCACCATCGGAG*C* |  |
| S12 F | 173-185 | *TCGAG*CTCGCCCGATGGTGCTCGTGGGCTTCCTCTGGGGGGGACTGTGA*A* | pAcGFP1-C1 |
| S12 R |  | *AGCTT*TCACAGTCCCCCCCAGAGGAAGCCCACGAGCACCATCGGGCGAG*C* |  |
| S13 F | 174-185 | *TCGAG*CTCCGATGGTGCTCGTGGGCTTCCTCTGGGGGGGACTGTGA*A* | pAcGFP1-C1 |
| S13 R |  | *AGCTT*TCACAGTCCCCCCCAGAGGAAGCCCACGAGCACCATCGGAG*C* |  |
| S14 F | 174-184 | *TCGAG*CTCCGATGGTGCTCGTGGGCTTCCTCTGGGGGGGATGA*A* | pAcGFP1-C1 |
| S14 R |  | *AGCTT*TCATCCCCCCCAGAGGAAGCCCACGAGCACCATCGGAG*C* |  |
| S15 F | 175-185 | *TCGAG*CTATGGTGCTCGTGGGCTTCCTCTGGGGGGGACTGTGA*A* | pAcGFP1-C1 |
| S15 R |  | *AGCTT*TCACAGTCCCCCCCAGAGGAAGCCCACGAGCACCATAG*C* |  |
| S16 F | 175-184 | *TCGAG*CTATGGTGCTCGTGGGCTTCCTCTGGGGGGGATGA*A* | pAcGFP1-C1 |
| S16 R |  | *AGCTT*TCATCCCCCCCAGAGGAAGCCCACGAGCACCATAG*C* |  |

**Table S3 Oligos used in this study 2**

| Oligos | Region (aa) | Sequences (5’-3’) | Vectors |
| --- | --- | --- | --- |
| T1 F | 174-183 | *TCGAG*CTCCGATGGTGCTCGTGGGCTTCCTCTGGGGGTGA*A* | pAcGFP1-C1 |
| T1 R |  | *AGCTT*TCACCCCCAGAGGAAGCCCACGAGCACCATCGGAG*C* |  |
| T2 F | 174-182 | *TCGAG*CTCCGATGGTGCTCGTGGGCTTCCTCTGGTGA*A* | pAcGFP1-C1 |
| T2 R |  | *AGCTT*TCACCAGAGGAAGCCCACGAGCACCATCGGAG*C* |  |
| T3 F | 174-181 | *TCGAG*CTCCGATGGTGCTCGTGGGCTTCCTCTGA*A* | pAcGFP1-C1 |
| T3 R |  | *AGCTT*TCAGAGGAAGCCCACGAGCACCATCGGAG*C* |  |
| T4 F | 174-180 | *TCGAG*CTCCGATGGTGCTCGTGGGCTTCTGA*A* | pAcGFP1-C1 |
| T4 R |  | *AGCTT*TCAGAAGCCCACGAGCACCATCGGAG*C* |  |

**Table S4 Oligos used in this study 3**

| Oligos | Region (aa) | Sequences (5’-3’) | Vectors |
| --- | --- | --- | --- |
| 189 F | 189-197 | *TCGAG*CTGTGGGCCTCGTGTTTCTGATCCTGCTCTGA*A* | pAcGFP1-C1 |
| 197 R |  | *AGCTT*TCAGAGCAGGATCAGAAACACGAGGCCCACAG*C* |  |
| 198 F | 198-204 | *TCGAG*CTCCGGTGCTCCGGGAGTCCGTCTGA*A* | pAcGFP1-C1 |
| 204 R |  | *AGCTT*TCAGACGGACTCCCGGAGCACCGGAG*C* |  |
| C1 F | 190-197 | *TCGAG*CTGGCCTCGTGTTTCTGATCCTGCTCTGA*A* | pAcGFP1-C1 |
| C1 R |  | *AGCTT*TCAGAGCAGGATCAGAAACACGAGGCCAG*C* |  |
| C2 F | 189-196 | *TCGAG*CTGTGGGCCTCGTGTTTCTGATCCTGTGA*A* | pAcGFP1-C1 |
| C2 R |  | *AGCTT*TCACAGGATCAGAAACACGAGGCCCACAG*C* |  |
| C3 F | 190-196 | *TCGAG*CTGGCCTCGTGTTTCTGATCCTGTGA*A* | pAcGFP1-C1 |
| C3 R |  | *AGCTT*TCACAGGATCAGAAACACGAGGCCAG*C* |  |
| C4 F | 191-196 | *TCGAG*CTCTCGTGTTTCTGATCCTGTGA*A* | pAcGFP1-C1 |
| C4 R |  | *AGCTT*TCACAGGATCAGAAACACGAGAG*C* |  |
| C5 F | 190-195 | *TCGAG*CTGGCCTCGTGTTTCTGATCTGA*A* | pAcGFP1-C1 |
| C5 R |  | *AGCTT*TCAGATCAGAAACACGAGGCCAG*C* |  |
| C6 F | 191-195 | *TCGAG*CTCTCGTGTTTCTGATCTGA*A* | pAcGFP1-C1 |
| C6 R |  | *AGCTT*TCAGATCAGAAACACGAGAG*C* |  |
| C7 F | 192-195 | *TCGAG*CTGTGTTTCTGATCTGA*A* | pAcGFP1-C1 |
| C7 R |  | *AGCTT*TCAGATCAGAAACACAG*C* |  |

**Table S5. Primers used in alanine mutagenesis assays**

| Primers | Positions | Sequences (5’-3’) |
| --- | --- | --- |
| M1 F | ^174^P/A | GACTGCCGCTACCCCGAAGACCGC**GCG**ATGGTGCTCGTGGGCTTCCTCTG |
| M1R |  | CAGAGGAAGCCCACGAGCACCAT**CGC**GCGGTCTTCGGGGTAGCGGCAGTC |
| M2 F | ^177^L/A | GTACCCCGAAGACCGC**GCG**ATGGTG**GCC**GTGGGCTTCCTCTGGGGGGGACTG |
| M2 R |  | CAGTCCCCCCCAGAGGAAGCCCAC**GGC**CACCAT**CGC**GCGGTCTTCGGGGTAC |
| M3 F | ^181^L/A | CGC**GCG**ATGGTG**GCC**GTGGGCTTC**GCC**TGGGGGGGACTGCTCCTGCTGGTG |
| M3 R |  | CACCAGCAGGAGCAGTCCCCCCCA**GGC**GAAGCCCAC**GGC**CACCAT**CGC**GCG |
| M4 F | ^185^L/A | **CC**GTGGGCTTC**GCC**TGGGGGGGA**GCG**CTCCTGCTGGTGGGCCTCGTGTTT |
| M4 R |  | AAACACGAGGCCCACCAGCAGGAG**CGC**TCCCCCCCA**GGC**GAAGCCCAC**GG** |
| M5 F | ^191^L/A | GGGGGGGA**GCG**CTCCTGCTGGTGGGC**GCC**GTGTTTCTGATCCTGCTC |
| M5 R |  | GAGCAGGATCAGAAACAC**GGC**GCCCACCAGCAGGAG**CGC**TCCCCCCC |
| M6 R | ^195^I/A | GC***GGTACC***TCAGGGAAACACGACGGACTCCCGGAGCACCGGGAGCAG**GGC**CAG |
| M9 R | ^200^L/A | GC***GGTACC***TCAGGGAAACACGACGGACTCCCG**GGC**CACCGG**GGCCGCGGC**CAG |
| M11 F | ^186^L/A | C**GCC**TGGGGGGGA**GCGGCC**CTGCTGGTGGGC**GCC**GTGTTT**GCGGCCGCG** |
| M11 R |  | **CGCGGCCGC**AAACAC**GGC**GCCCACCAGCAG**GGCCGC**TCCCCCCCA**GGC**G |
| M12 F | ^187^L/A | C**GCC**TGGGGGGGA**GCGGCCGCG**CTGGTGGGC**GCC**GTGTTT**GCGGCCGCG** |
| M12 R |  | **CGCGGCCGC**AAACAC**GGC**GCCCACCAG**CGCGGCCGC**TCCCCCCCA**GGC**G |
| M13 F | ^188^L/A | C**GCC**TGGGGGGGA**GCGGCCGCGGCG**GTGGGC**GCC**GTGTTT**GCGGCCGCG** |
| M13 R |  | **CGCGGCCGC**AAACAC**GGC**GCCCAC**CGCCGCGGCCGC**TCCCCCCCA**GGC**G |

**Table S6. Oligos used in alanine mutagenesis assays**

| Oligos | Positions | Sequences (5’-3’) |
| --- | --- | --- |
| M7 |  | C**GCC**GTGTTTCTG**GCCGCG**CTCCCGGTGCTCCGGGAGTCCGTCGTGTTTCCCTGA |
| M8 |  | GGGC**GCC**GTGTTTCTG**GCCGCGGCC**CCGGTGCTCCGGGAGTCCGTCGTGTTTCCCTGA |
| M10 |  | GCTGGTGGGC**GCC**GTGTTT**GCGGCCGCGGCC**CCGGTG**GCG**CGGGAGTCCGTCGTGTTTCCCTGA |
